# Microbiome modulation for sustainable crop production and climate resilience

**DOI:** 10.1093/sumbio/qvag032

**Published:** 2026-07-28

**Authors:** Malek Marian, Ioannis Stringlis, Eleonora Rolli, Christopher James Barnes, Stéphane Compant, Angela Sessitsch

**Affiliations:** AIT Austrian Institute of Technology, Bioresources Unit, Konrad Lorenz Strasse 24, 3430 Tulln an der Donau, Austria; Laboratory of Plant Pathology, Agricultural University of Athens, 75 Iera Odos st., 11855 Athens, Greece; Department of Food, Environmental and Nutritional Sciences, University of Milan, via Celoria 2, 20133 Milan, Italy; Department of Agroecology, Faculty of Technical Sciences, Aarhus University, Forsoegsvej 1, Slagelse, 4200, Denmark; Centre for Evolutionary Hologenomics, The Globe Institute, Faculty of Health, University of Copenhagen, Oester Farimagsgade 5, Copenhagen 1014, Denmark; AIT Austrian Institute of Technology, Bioresources Unit, Konrad Lorenz Strasse 24, 3430 Tulln an der Donau, Austria; AIT Austrian Institute of Technology, Bioresources Unit, Konrad Lorenz Strasse 24, 3430 Tulln an der Donau, Austria

**Keywords:** plant disease control, plant growth promotion, bioinoculant, rhizosphere engineering, microbial ecology

## Abstract

The microbiome is fundamental to plant performance in agroecosystems, influencing primary productivity and climate resilience. Microbiome modulation refers to the targeted manipulation (or steering) and optimization of microbiota features, including taxonomic structure, diversity, composition, assembly dynamics, stability, functional capacity, interactions, and network architecture. Here, we review the current state-of-the-art knowledge and strategies used for microbiome modulation, encompassing biological interventions such as bacteria, fungi, protists, nematodes, and phages, as well metabolites, compounds, and nutrients derived from plants. We further discuss emerging tools and strategies for next-generation microbiome modulation, including function-oriented, multitrophic defined microbial communities assembled based on ecological traits and interactions across multiple trophic levels to enable their establishment and function within the phytobiome; temperate phages; microbiome transplantation and breeding; and functional synbiotics, defined as combinations of beneficial microorganisms and compounds that improve microbiome health and function. In addition, we highlight key knowledge gaps and research priorities for advancing precision microbiome modulation. Addressing current challenges will require integrated frameworks combining experimental validation *in planta* and reductionist approaches with *in silico* modeling and multiomics analyses to better predict, design, and sustain beneficial plant-microbiome outcomes. Overall, microbiome modulation represents a paradigm shift in advancing sustainable and climate-resilient agri-food systems.

**Sustainability Statement**

Microbiome modulation offers a promising strategy for enhancing sustainable crop production and climate resilience. This review primarily aligns with the United Nations Sustainable Development Goals (SDG 2: Zero Hunger) by advancing mechanistic understanding and methodological approaches, and by enabling scalable and transnational applications for the development of innovative biological interventions in agriculture. Ultimately, microbiome-based applications can support environmentally friendly, nature-based farming practices, contributing to One Health and long-term global food security. In addition, this work is relevant to SDG 13 (Climate Action), SDG 15 (Life on Land), and SDG 12 (Responsible Consumption and Production).

## Introduction

The microbiome is fundamental to plant performance in agroecosystems, influencing key processes related to sustaining primary productivity and climate resilience (Singh et al. 2020, Trivedi et al. 2022). Consequently, there is growing interest in the use of biological interventions, including microbial inoculants, soil fauna, and phages, as well metabolites, compounds, and nutrients derived from plants, to deliberately shape the native microbiome features and enhance plant performance (Pascale et al. 2020, Berg et al. 2021, Li et al. 2024a, Luan et al. 2025, Salehimoghaddam et al. 2026). Recently, the concept of microbiome modulation (Box 1) was introduced as a distinct and novel mode of action for biological interventions, particularly for microbial inoculants (Berg et al. 2021). Modulation strategies may include the targeted enrichment of beneficial microbial taxa and the depletion of the pathogen-facilitating ones (i.e. the pathobiome). Numerous studies have demonstrated that biological interventions not only can induce direct effect on plant growth and health but also shift the native microbiome across plant-associated compartments (rhizosphere, phyllosphere, and endosphere) (Pascale et al. 2020, Berg et al. 2021, Li et al. 2024a, Luan et al. 2025, Salehimoghaddam et al. 2026), often enriching taxa linked to nutrient mobilization, pathogen suppression, and abiotic stress mitigation. Microbiome-based applications have become increasingly sophisticated (Compant et al. 2025) and may encompass specific microbiome modulators, including defined microbial communities (DMCs) (Koch et al. 2026), as well as data-driven or precision-guided approaches (Kostic et al. 2024). These advances reflect a transition from single-strain microbials toward systems-oriented strategies aimed at steering key community features, including taxonomic structure, diversity, composition, assembly dynamics, stability, functional capacity, interactions, and network architecture.

Box 1. Glossary of key terms.

**Table:** 

Term	Definition
**Microbiota/microbial community**	The collective (living) microorganisms in a habitat including bacteria, archaea, and eukaryotic microorganisms (fungi, protists, and algae).
**Microbiome**	The genetic and structural components (including viruses and phages), metabolites, and collective activities associated with the microbiota and its environment.
**Phytobiome**	All the biotic components associated with and interacting with the host plant, including plants themselves, microorganisms, fauna (nematodes, arthropods, and earthworms), and viruses.
**Pathobiome**	The microbial taxa, or interactions among these taxa with other organisms and host plant, that are associated with a reduced host health status.
**Nemato-protective microbiome**	The microorganisms that assist nematodes during survival and development in hosts.
**Defined microbial community (DMC)**	Consortia consisting of multiple cultured and well-described microbial strains that are deliberately assembled in the laboratory (Koch et al. 2026).
**Keystone taxa**	Specific members of the microbiome exerting a disproportionately large impact on ecosystem structure, stability, and function relative to their abundance.
**Microbiome modulation**	The targeted manipulation (or steering) and optimization of microbiota to enhance agricultural productivity and/or ecosystem resilience.
**Phytobiome modulation**	The targeted manipulation (or steering) and optimization of microbial, faunal, and viral communities to enhance agricultural productivity and/or ecosystem resilience.
**Synbiotic**	The combination of probiotic (live microorganism that may confer benefits to the host) and prebiotic (substrate which is selective for the proliferation or maintenance of the probiotic within the target microbiome), which together improve the health and function of a microbiome.

This review focuses on recent advances in microbiome modulation strategies. We synthesize the current state-of-the-art knowledge and strategies used for microbiome modulation, discuss emerging tools and strategies for next-generation microbiome modulation, and highlight key knowledge gaps and research priorities for advancing precision microbiome modulation.

## Bacterial and fungal inoculants as modulators of the microbiome

Bacteria and fungi are the most widely used biological components of microbiome-modulation strategies in plant-associated systems. Rather than acting exclusively through direct plant growth promotion, bacterial and fungal inoculants frequently function as ecological regulators that reshape the native microbiome, thereby influencing composition, interaction networks and ecosystem functioning (Table 1, Li et al. 2024a, Francioli et al. 2025). Microbiome modulation by bacteria and fungi produces measurable ecological changes across multiple biological scales. At the community level, inoculation can shift taxonomic composition and enhance microbial network connectivity. At the functional level, modulation may improve nutrient cycling, pathogen suppression, and stress tolerance. At the ecosystem level, these changes can translate into increased productivity stability and soil health. However, microbiome responses remain context dependent. Soil physicochemical properties, plant genotypes, and initial microbiome composition strongly influence inoculant success. Recent analyses have shown that native microbiomes compatible with inoculants significantly enhance inoculation outcomes, reinforcing the need for targeted-based approaches (Li et al. 2024a). The authors performed a meta-analysis of 335 studies showing that, overall, inoculants positively influence soil microbial biomass, with effects enhanced by fertilizers and native strains but weakened under environmental stress. Although they did not change overall microbial diversity, inoculants can often significantly shift community structure and bacterial composition, reducing network complexity while increasing network stability. Higher initial soil nutrient levels generally further amplified benefits on fungal biomass, actinomycetotal biomass, microbial biomass carbon, and microbial biomass nitrogen.

**Table 1:** Recent studies on bacterial and fungal inoculant as modulators of the microbiome.

Bacterial or fungal inoculant	Plant species/system	Plant performance outcome	Impact on microbiome (structure/function)	Reference(s)
Mixed microbial inoculants (meta-analysis)	Multiple systems	Context-dependent efficacy	Increased microbial biomass Shifted community composition Reduced network complexity and increased stability No change in diversity	Li et al. (2024a)
DMC (*Bacillus, Paenibacillus*, and *Pantoea* spp.)	Wax gourd (*Benincasa hispida* L.)	Decreased disease incidence of *Phytophthora capsici*	Enriched beneficial genera like *Bacillus, Delftia*, and *Pseudomonas* in the wax gourd phyllosphere	Ali et al. (2026)
Core root DMC	Tomato	Increased salinity tolerance	Influenced the microbial network of the natural microbiome by increasing the average connectivity	Schmitz et al. (2022)
*Paraburkholderia fungorum* strain BRRh-4 and *Delftia* sp. strain BTL-M2	Rice	Increased plant growth and grain yield under 50% reduced NPK fertilization	Increased bacterial diversity in roots Altered bacterial community composition, including enrichment of *Sphingomonas, Novosphingomonas*, and *Massilia*, particularly following BRRh-4 inoculation	Islam et al. (2023)
AMF and *Devosia*	Maize	Promoted plant growth, nitrogen uptake, mycorrhization	Enhanced nutrient-related microbiome functions	Zhang et al. (2024)
*Rhizophagus irregularis* (AMF)	Maize	Increased leaf phosphorus; genotype-dependent responses	Altered bacterial gene expression; higher fungal diversity Stimulated B-vitamin biosynthesis; enriched AMF-associated taxa	Wright et al. (2025)
*Bacillus velezensis* ES2-4	Soybean	Improved resistance to *Botrytis*	ES2-4 application restored bacterial richness, particularly within the *Alphaproteobacteria* and *Streptomyces* lineages, while reducing the relative abundance of fungal pathogens. ES2-4 inoculation led to the upregulation of antifungal metabolites (e.g. oxalic acid and eicosane) in root exudates	Chen et al. (2025b)
DMC *(Trichoderma, Pseudomonas*, and *Bacillus* spp.)	Maize	Enhanced plant growth; improved micronutrient uptake; improved drought tolerance	Increased relative abundance of bacterial taxa (e.g. *Myxococcota, Bacteroidota*, and *Pseudomonadota*) Enriched fungal taxa (e.g. *Talaromyces* and *Trichoderma*) Increased the abundance of genes related to microbial siderophores and the root exudation of benzoxazinoids	Francioli et al. (2025)

Bacterial and fungal inoculants can also promote the beneficial functions of plant-associated microbiome. Species belonging to *Pseudomonas* and *Bacillus* provide well-documented examples of microbiome modulation beyond direct plant stimulation. For instance, *Bacillus velezensis* ES2-4 modulated root exudation and microbiome remodeling to enhance soybean resistance against grey mold (Chen et al. 2025b). Francioli et al. (2025) further showed that a DMC of *Pseudomonas* sp. RU47, *Bacillus atrophaeus* ABi03, and *Trichoderma harzianum* OMG16, effectively enhanced maize growth and fitness across both intensive and extensive farming systems. Inoculation with this DMC improved iron uptake, modulated hormonal balance, and enhanced reactive oxygen species detoxification, supporting drought adaptation. However, the tested DMC also reshaped rhizosphere bacterial and fungal communities and enriched microbial genes involved in siderophore production and antimicrobial lipopeptides. Moreover, DMCs enhanced wax gourd plant growth and suppressed *Phytophthora capsici* by microbiome modulation via competitive exclusion, enriching beneficial genera like *Bacillus, Delftia*, and *Pseudomonas* in the phyllosphere (Ali et al. 2026). Schmitz et al. (2022) demonstrated that designed DMC based on core bacterial taxa enhanced salinity stress tolerance of tomato, and this improvement was attributed to the restructuring of the rhizosphere microbiome. Furthermore, inoculation of rice with *Paraburkholderia* and *Delftia* improved rice growth, grain yield, and root and rhizosphere bacterial diversity. Notably, these benefits were maintained under a 50% reduction in N, P, and K fertilizer inputs, highlighting the potential of microbiome modulation to reduce agrochemical dependence while sustaining productivity (Islam et al. 2023). Overall, these findings demonstrate that targeted DMCs can improve plant resilience and productivity under variable environmental and management conditions.

Fungi exert particularly strong influence on microbiome structure due to their filamentous growth and ability to span spatially heterogeneous environments. It has been shown that the Arbuscular Mycorrhizal Fungi (AMF)-associated bacterium *Devosia* synergistically acts with mycorrhiza on the plant root to strongly promote plant growth, nitrogen uptake, and mycorrhization (Zhang et al. 2024). Using maize, Wright et al. (2025) showed that *Rhizophagus irregularis* alters bacterial gene expression in rhizosphere and bulk soil, without shifting community composition, and significantly increases fungal richness and evenness. They identified genotype-specific effects of AMF on microbial diversity and function and found that fungal colonization stimulates microbial vitamin B biosynthesis. They also linked elevated plant leaf phosphorus levels under AMF colonization with changes in root gene expression and increased abundance of AMF-stimulated rhizosphere bacterial taxa.

Fungi additionally influence microbiome spatial organization since hyphal networks function as dispersal corridors that enable bacterial movement—a mechanism often termed “fungal highways” (Vieira et al. 2025). Experimental microcosm studies have revealed that beneficial bacteria can migrate along fungal hyphae to colonize plant roots more efficiently, increasing microbial connectivity and community stability under fluctuating environmental conditions. For example, a *B. velezensis* strain when co-inoculated with AMF fungus *R. irregularis*, uses fungal hyphae as highways for soil invasion and colonizing new plants (Anckaert et al. 2024). The fungus was found to expand nutrient access through hyphal networks, while the bacterium mineralizes nutrients and suppresses pathogens, collectively reshaping microbiome structure toward beneficial functional guilds. *Bacillus velezensis* colonizes the entire mycelial network more efficiently than roots. The lipopeptide surfactin plays key roles in the chemical ecology of the interaction and the microbial partnership enhances the systemic resistance of tomato against *Botrytis* (Anckaert et al. 2024). Table 1 summarizes recent studies on modulation of the plant microbiome after application of bacterial or fungal inoculants.

## Protists and nematodes as modulators of the microbiome

Protists, unicellular microbial eukaryotes, are major predators of soil bacteria and fungi and play a central role in regulating soil food webs. Although they do not directly increase plant biomass, they can exert indirect effects on plant performance by reshaping microbiome composition and function and by promoting nutrient release, thereby enhancing crop productivity (de Gea et al. 2025, Santoyo et al. 2025). For example, Guo et al. (2024) demonstrated that *Colpoda lenta* grazing enriched plant growth-promoting rhizobacterial taxa (*Pseudomonas* and *Sphingomonas*), and strengthened microbial interactions associated with enhanced biofilm formation. These shifts promoted phosphorus mobilization in the rhizosphere and ultimately improved cucumber performance. More recently, phagotrophic protists were found to reduce the dominance of highly abundant bacterial taxa while increasing the diversity of nitrogen-fixing bacteria, primarily by stimulating rare taxa (Zeng et al. 2026). These microbiome shifts were associated with altered plant hormone dynamics, including increased cytokinin and reduced ethylene levels in peanut roots, which in turn regulated the expression of genes involved in rhizobial infection, nodule organogenesis, and bacteroid differentiation. Inoculation of maize plants with DMC consisting of 18-member protists enriched specific rhizosphere bacterial taxa, including members of the *Oxalobacteriaceae* and *Burkholderiaceae* that were originally associated with protist cultures (Taerum et al. 2024). These protist-associated bacteria conferred protection against growth-suppressive soil microorganisms, thereby promoting plant performance (Taerum et al. 2025). Mechanistically, this interaction was linked to tryptophan-dependent biosynthesis of the auxin indole-3-acetic acid (IAA), which underpins the protist−bacteria interaction and its plant-beneficial effects (Patel et al. 2025).

Accumulating evidence further shows the impact of protist-driven microbiome changes on plant health (Guo et al. 2022, Guo et al. 2024, Song et al. 2025). For example, *Cercomonas lenta* grazing on rhizosphere bacteria enriched other disease suppressive bacterial taxa (e.g. *Bacillus* spp.). The trophic interaction between the predatory protists and *Bacillus* spp. increased the relative abundance of non-ribosomal peptide synthetase gene, encoding antimicrobial lipopeptides (e.g. bacillomycin), within the microbiome, ultimately reducing the disease incidence of Fusarium wilt on banana (Guo et al. 2022). Furthermore, the predatory protist *Colpoda* was shown to reduce the incidence of tomato bacterial wilt both through direct consumption of the pathogen and indirect microbiome-mediated effects. Specifically, *Colpoda* grazing enriched pathogen-suppressive bacterial taxa, including *Pseudomonas, Lysobacter*, and *Streptomyces*, while simultaneously depleting pathobiome-associated members such as *Arthrobacter* and *Chitinophaga* (Guo et al. 2024). These studies highlight the impact of protist predation on microbiome features and thereby on plant growth and health, indicating the potential of protists to engineer more robust and plant-beneficial microbiomes. However, the microbial functions or traits underlying the effect of protists on the microbiome features for improved plant growth and health remain unclear. Future research should identify the microbial traits that determine susceptibility or resistance to protist grazing and elucidate how protist-driven selection reshapes microbiome assembly, metabolic cross-feeding, and disease-suppressive functions across different soil and cropping systems. Integrating shotgun metagenomics, targeted metatranscriptomics, metabolomics, DMCs, and mutant-based approaches will be essential to identify microbial functional traits and interaction networks underlying protist-mediated microbiome modulation and plant-beneficial outcomes.

Free-living nematodes, which are mostly beneficial in promoting plant growth and health, include bacterivores and fungivores that graze upon bacteria and fungi, respectively (Li et al. 2024b). These feeding preferences can shape the structure of rhizosphere microbiome, with consequent effect on plant growth and disease outcome as well as soil multifunctionality (Jiang et al. 2020, 2023b, Li et al. 2024b, Zheng et al. 2026). For example, nematode predation enriched certain groups, such as *Actinomycetota, Chloroflexota*, and *Bacillota* (formerly known as *Actinobacteria, Chloroflexi*, and *Firmicutes*) and strengthened microbial network connectivity (*Actinomycetota* and *Pseudomonadota*). These changes were associated with an increased abundance of phosphate-solubilizing bacteria that facilitated phosphorus cycling, leading to greater phosphorus uptake and biomass of wheat (Jiang et al. 2023b). Recent studies further highlight the importance of trophic buffering, a process by which predation-mediated interactions maintain beneficial microbial functions by stabilizing key microbial populations within complex communities (Huo et al. 2026). In this study, the authors demonstrated that in the presence of nematodes, the plant growth-promoting effect of *Streptomyces* on *Arabidopsis thaliana* were sustained due to nematodes selectively grazing on competing microbial taxa, reshaping the native community and alleviating competitive constraints on *Streptomyces* (Huo et al. 2026). Furthermore, bacterivorous nematodes (*Protorhabditis*) can change the microbiome composition and functions by favoring antagonistic bacteria, such as *Bacillus*, and the combined application of *Protorhabditis* and *Bacillus* led to a high protection level against tomato bacterial wilt disease incidence (Xu et al. 2024). Future research should determine which microbial traits confer susceptibility or resistance to nematode grazing and how these trophic interactions promote disease suppression, nutrient cycling, and other emergent microbiome functions across different soil and cropping systems.

Plant-parasitic nematodes, including the root-knot nematode *Meloidogyne* spp. can substantially alter the microbiome structure and functions following infestation (Li et al. 2024b). Emerging evidence further suggests that specific microbial groups may facilitate nematode survival and infection, forming a “nemato-protective microbiome” that supports parasitism (Topalović and Vestergård 2021, Topalović et al. 2023, Li et al. 2024b). For example, nematode infestation has been linked to the enrichment of nitrogen-fixing bacteria, including *Rhizobiales, Betaproteobacteriales* (or *Burkholderiales*), and *Rhodobacterales* taxa, which may contribute functionally to nematode parasitism (Li et al. 2023), by for example enabling the nematodes to evade plant immune responses (Topalović and Vestergard 2021). In addition, the composition of nematode-attached microbiomes, including taxa such as *Algoriphagus, Pedobacter*, and *Bdellovibrio*, has been associated with *Meloidogyne* spp. activity (Topalović et al. 2023). However, it remains unclear whether members of this nematode-protective microbiome can be selectively targeted to reduce nematode fitness while preserving beneficial activities of the native microbiome. Addressing this question could support the development of microbial bioinoculants that disrupt nematode-associated microbial functions, interactions, and networks, thereby improving the biological control of plant-parasitic nematodes.

## Phages as modulators of the microbiome

Phages, viruses that infect bacteria, can generally affect bacterial populations, (i) directly through the phage lytic lifestyle where the lysis of bacteria results in bacterial community turnover and serves as a nutrient source and (ii) indirectly through phage lysogenic lifestyle that could act as a novel genetic source (Pratama et al. 2020).

Lytic (virulent) phages are traditionally applied in agriculture to replicate and kill bacterial pathogens of crops (Pratama et al. 2020). However, recent studies have also shown that lytic phages can indirectly shape the microbiome features (composition and function). In the phyllosphere, Morella et al. (2018) demonstrated the impact of phages on the associated bacterial community composition and abundance, including *Pseudomonadaceae* and *Enterobacteriaceae* taxa. More recently, Forero-Junco et al. (2022) used viral metagenomics to characterize the phage communities in the wheat phyllosphere. Their results revealed an active community of novel phages predicted to infect the dominant bacterial families, including *Pseudomonadaceae* and *Xanthomonadaceae*. Genomic evidence recently revealed an underexplored infection mode known as pseudo-lysogeny, in which lytic phages coexist with their bacterial hosts without integrating into the chromosome or initiating active replication (Perfilyev et al. 2025). This suggests that phages may persist within the microbiome and contribute to horizontal gene transfer, host evolution, and modulation of host physiology, extending their role beyond purely antagonistic, host-killing interactions (Perfilyev et al. 2025).

Phage-driven microbiome modulation can result in an improved disease suppression, including against bacterial wilt of tomato (Wang et al. 2024b), bacterial canker of kiwifruit (Hu et al. 2025b), and bacterial leaf blight of rice (Jiang et al. 2023a). Phages can mediate these effects by freeing ecological niche space, triggering community reassembly following pathogen reduction, and releasing metabolites from lysed bacterial hosts (Yang et al. 2023b, Wang et al. 2024b, Franco Ortega et al. 2024, Lee et al. 2024). For example, phage application increased the diversity of rhizosphere microbiota and enriched disease-suppressive taxa belonging to *Actinomycetota* (*Streptomyces* and *Nocardioides*). These taxa acted synergistically with phages to suppress *Ralstonia solanacearum* through complementary mechanisms involving resource and interference competition (Wang et al. 2024b). Debray et al. (2025) further demonstrated a critical role for phage communities in limiting the population growth of *Pseudomonas syringae* via microbiome modulation. Plants harboring phage-depleted microbiomes were more susceptible to infection, an effect that could not be attributed to direct phage impacts on either the pathogen or the tomato host. However, other studies have found that phage treatments had no drastic effect on the diversity and composition of the microbiome even in the field (Magar et al. 2024, Holtappels et al. 2025). These contrasting findings indicate that phage-mediated microbiome modulation is context-dependent, both influenced by and capable of reshaping the ecological and functional properties of the native microbiome. The effects of phages on bacterial community structure, diversity, and abundance across plant compartments are well documented (Pratama et al. 2020), yet their roles in modulating population dynamics and molecular interactions within the endosphere remain poorly understood.

Temperate phages, which integrate into bacterial host genomes and replicate as prophages, can confer fitness benefits to their hosts, including defense and ecological processes (Salehimoghaddam et al. 2026). These phages can also play a role in shaping microbiome features and plant−microbiome interactions (Basso et al. 2025, Zhong et al. 2025). For example, Basso et al. (2025) detected and characterized a double-stranded DNA prophage in the plant growth-promoting rhizobacteria *Pseudomonas simiae* WCS417. By comparing the GFP-labeled wild-type strain with a prophage-deletion mutant (Δphage) in root colonization, metabolomics, imaging, and thermal stress assays, the authors demonstrated that prophage presence influenced root exudate composition, root architecture, and shoot biomass under heat stress, while having little effect on bacterial colonization ability.

To date, most microbe- and microbiome-based strategies for plant health enhancement have relied on bioaugmentation with antagonistic organisms that directly inhibit pathogens and pests. Pathogens and pests are known to manipulate the plant environment to create a favorable niche or to deactivate immune responses. Growing evidence further indicates that they can modulate the plant microbiome features and rhizosphere metabolism for successful (co)infection (Snelders et al. 2022, Xu et al. 2026a). Certain members of this modulated microbiome, associated with a reduced health status of the host plant, are collectively referred to as ‘‘pathobiomes’’ (Alfaro‐García et al. 2026). Targeted modulation of the pathobiome members using phages could therefore provide a promising avenue for precision microbiome modulation (Hessler et al. 2025), offering the potential to suppress diseases and enhance plant stress resilience while minimizing disruption to the native microbiome. Alternatively, phages infecting beneficial plant growth-promoting bacteria were able to modulate the rhizosphere microbiome disease suppressiveness against *R. solanacearum* through the activation of antimicrobial activity (Yang et al. 2026). The authors showed that exposing *Stenotrophomonas maltophilia* to phages in the tomato rhizosphere increases the disease suppressiveness of the microbiome by stabilizing bacterial diversity and facilitating pathogen suppression by other native species. Despite these advances, further studies are still required to validate the stability, efficacy, and translational potential of phage-based approaches under field conditions.

Beyond direct pathogen and indirect pathobiome targeting, innovative phage-based strategies are emerging. For example, Zhang et al. (2026c) developed a microbiome modulation approach that harnesses phoresy, i.e. an ecological interaction in which one organism disperses via association with a more mobile partner, within phage-bacteria systems. In this study, hitchhiking phage cocktails onto carrier biocontrol bacteria (*Bacillus subtilis*) were shown to enhance the eradication of pathogens’ (*Pantoea agglomerans* and *P. syringae*) biofilm in the soybean phyllosphere. This phoresy system enabled efficient pathogen lysis while simultaneously facilitating colonization by beneficial bacteria. The establishment of foliar-beneficial symbionts reshaped the metabolic activity (e.g. biosynthesis of flavonoid and tropane alkaloid metabolites) of the phyllosphere microbiome and significantly improved overall plant disease resistance.

## Microbiome-modulating metabolites, compounds, and nutrients

Plants actively shape their associated microbiomes through the release of metabolites, signaling compounds, and nutrients into the rhizosphere, creating a dynamic interface between roots and soil microbial communities (Bakker et al. 2020, Pascale et al. 2020, Uribe-Acosta et al. 2025). Through the “rhizosphere effect,” root exudates strongly influence microbial composition, abundance, and activity. These exudates include both primary metabolites (e.g. sugars, amino acids, and organic acids) and specialized metabolites (e.g. flavonoids, coumarins, terpenoids, alkaloids, peptides, and phenolics). Beyond serving as carbon sources or antimicrobials, these compounds can act as ecological filters and signaling molecules that regulate microbial recruitment and activity. Importantly, plant metabolites can also function as behavioral cues, shaping microbial chemotaxis, motility, and colonization efficiency. Together, these processes enable plants to enrich beneficial microorganisms, suppress pathogens, and enhance ecosystem resilience (Pascale et al. 2020, Robert et al. 2025).

One of the best-studied groups of microbiome-modulating metabolites are flavonoids (Table 2). Beyond mediating symbiotic interactions, particularly with nitrogen-fixing bacteria and mycorrhizal fungi, recent studies demonstrated that flavonoids can enhance plant defense and growth while simultaneously shaping beneficial microbial communities. For example, in pepper, the flavonoid apigenin enhances resistance to *P. capsici* while enriching disease-suppressive microorganisms (Liao et al. 2025). In poplar, flavonoids such as tricin and apigenin recruited beneficial *Pseudomonas*, improving growth and nitrogen acquisition under nutrient-poor conditions (Wu et al. 2025b). In maize, root-derived flavones selectively enriched *Oxalobacteraceae*, which contribute to enhanced nitrogen uptake and plant performance (Yu et al. 2021a). In legumes, isoflavones regulated rhizosphere microbiota by mediating symbiotic interactions and influencing microbial colonization dynamics (Aoki et al. 2024). In addition, flavonoid-mediated interspecific interactions can shape microbiome recruitment; for instance, the flavonoid taxifolin released by potato onion roots promoted the recruitment of beneficial *Bacillus* spp. in tomato rhizospheres, contributing to suppression of *Verticillium dahliae* in intercropping systems (Zhou et al. 2023b).

**Table 2:** Chemical drivers of microbiome modulation: key metabolites, compounds, and nutrients shaping plant−microbiome interactions.

Class of compounds	Representative metabolites/compounds	Plant species/system	Main activity	Impact on microbiome features (structure/function)	Reference(s)
Flavonoids	Apigenin; tricin; flavones; isoflavones; taxifolin	Pepper; poplar; maize; legumes; tomato intercropping	Antimicrobial, signaling, symbiosis support	Recruited beneficial taxa such as *Pseudomonas, Bacillus*, and *Oxalobacteraceae* Promoted nitrogen uptake, growth and disease suppression	Yu et al. (2021a), Zhou et al. (2023b), Aoki et al. (2024), Liao et al. (2025), Wu et al. (2025b)
Phenolics/coumarins	4-hydroxycinnamic acid (4-HCA); coumarins	Rice; Arabidopsis; diverse plant communities	Metabolic signal, ecological filter, induced systemic resistance-associated signaling	Enriched beneficial Pseudomonadales Reshaped fungal communities and networks Modulated bacterial gene expression, motility, and root colonization Contributed to soil-borne legacy effects	Stringlis et al. (2018), Voges et al. (2019), Yu et al. (2021b), Vismans et al. (2022), Su et al. (2024), Li et al. (2026b)
Alkaloids, phytoalexins, and saponins	Amaryllidaceae alkaloids; α-tomatine; tomatidine; camalexin	Red spider lily *(Lycoris radiata* L’Hér); tomato; Arabidopsis	Antimicrobial defense and selective pressure	Selected tolerant/beneficial microbiota Enriched *Sphingomonadaceae* and *Sphingobium* Altered bacterial motility, chemotaxis, and colonization dynamics	Nakayasu et al. (2021), Koprivova et al. (2023), Nakayasu et al. (2023); (Zhou et al. 2024b), 2025)
Indole-derived defense compounds	Benzoxazinoids; HDMBOA-Glc; linalool-triggered pathway	Maize	Defense signaling and microbiome restructuring	Reshaped root and rhizosphere microbiota under field conditions Promoted resistance-associated microbiome states in subsequently grown plants	Cadot et al. (2021), Guo et al. (2025)
Terpenoids and sterol-related compounds	Cucurbitacin B; β-sitosterol; stigmasterol; cholesterol; steroidal glycoalkaloids/saponins	Melon; tomato	Antimicrobial, metabolic regulation, ecological filtering	Enriched *Enterobacter* and *Bacillus* Reshaped root-associated bacterial families such as *Comamonadaceae* and *Sphingomonadaceae*	Zhong et al. (2022), Der et al. (2024), Guerrieri et al. (2025)
Glucosinolates	Indole glucosinolates and hydrolysis products	Arabidopsis	Defense metabolites and selective filters	Altered rhizoplane, endosphere and rhizosphere community composition Selected bacteria tolerant to glucosinolate breakdown products	Kudjordjie et al. (2021), Basak et al. (2024), Chroston et al. (2024), Acuña et al. (2025), Russ et al. (2025)
Peptides	Plant small peptides; prosystemin-derived peptide	Tomato; conceptual rhizosphere signaling framework	Defense signaling and microbiome modulation	Restructured microbiome composition Enriched taxa associated with defense and resilience, including *Sphingobium, Sphingomonas, Massilia*, and *Brevundimonas*	Tan et al. (2024), Castaldia et al. (2025)
Amino acids	Glutamic acid; glutamine	Various plants/roots	Nutrient source, chemoattractant, colonization cue	Enriched taxa such as *Streptomyces* Regulated spatial colonization and prevented excessive rhizobacterial proliferation	Kim and Kwak (2022), Tsai et al. (2025)
Organic acids	Citrate; malate; oxalate; succinic acid; methylmalonic acid	Various plants; rice	Nutrient solubilization, recruitment signal	Shaped rhizosphere communities Recruited beneficial taxa such as *Pseudomonas* linked to phosphorus acquisition	Liu et al. (2024), Cesco et al. (2026)
Sugars and related compounds	Ribose; xylose; mannose; maltose; lactic acid; gluconolactone; ribitol; sucrose; trehalose; inositol	Tomato; maize; general rhizosphere recruitment	Prebiotic carbon sources; behavioral cues	Supported commensals over pathogens Shifted community structure across development Influenced colonization behavior through motility- and biofilm-related traits	Tian et al. (2021), Lopes et al. (2022), O’Banion et al. (2023), Sánchez-Gil et al. (2023), Wen et al. (2023)
Stress-induced mixed exudates	Organic acids, sugars and amino acids	Sanchi ginseng (*Panax notoginseng* (Burkill) FH Chen)	Stress-responsive exudation	Suppressed soil-borne pathogens while enriching beneficial taxa such as *Trichoderma, Bacillus* and *Streptomyces* Reversed negative plant–soil feedback	Luo et al. (2022)
Vitamins and fatty acids	Riboflavin; 3-hydroxyflavone; palmitic, oleic, linoleic and stearic acid	Tomato; Sanchi ginseng	Defense-associated signaling and nutritional selection	Restored suppressive microbiomes by enriching *Actinomycetota*/*Streptomyces* Selectively enriched *Mortierella* and promoted resilience/productivity	Yang et al. (2023a), Hu et al. (2026)

Phenolic compounds also contribute significantly to microbiome assembly. For instance, a genome-wide association study in rice revealed that the lignin precursor 4-hydroxycinnamic acid, produced via OsPAL02, drove the enrichment of beneficial *Pseudomonadales*, with loss of this pathway leading to microbiome dysbiosis and increased disease susceptibility (Su et al. 2024). Among phenolic compounds, coumarins have emerged as an important class of microbiome- shaping metabolites (Stringlis et al. 2018, Voges et al. 2019). Plant diversity strongly shapes soil microbial communities, particularly fungi, increasing diversity, altering composition, and enhancing stability, especially in nutrient-poor soils (Li et al. 2026b). These effects are often linked to shifts in root exudation, with coumarins emerging as key regulators that structure fungal communities, microbial networks, and functional profiles. Beyond their ecological role as selective metabolites, coumarins act as signaling molecules in plant−microbe interactions. In Arabidopsis, coumarin secretion induced by the beneficial bacterium *P. simiae* WCS417 modulated bacterial gene expression, reduced motility, and promoted root colonization while triggering systemic resistance in the host (Yu et al. 2021b). Similarly, coumarins were found to be central to the establishment of a soil-borne legacy effect, where pathogen-induced changes in root exudation reshaped the rhizosphere microbiome and enhanced disease resistance in subsequent plant generations via salicylic acid-dependent defenses (Vismans et al. 2022).

Alkaloids, phytoalexins, terpenoids, sterol-derived compounds, glucosinolates, and peptides are increasingly recognized as important drivers of microbiome assembly because they can directly inhibit pathogens while selectively enriching tolerant or beneficial microorganisms. In *Lycoris radiata*, Amaryllidaceae alkaloids (AAs) showed differential antimicrobial activity against endophytes and fungal pathogens, while AA-insensitive bacteria enhanced AA accumulation, generating a positive feedback loop that favored tolerant microbiota and improved protection (Zhou et al. 2024b). In tomato, the steroidal glycoalkaloid/saponin α-tomatine and its aglycone tomatidine shaped rhizosphere bacterial communities and enriched *Sphingomonadaceae*, whereas later work showed that tomato-associated *Sphingobium* strains possess a dedicated pathway to catabolize α-tomatine, explaining their selective enrichment in tomato roots (Nakayasu et al. 2021, 2023). The indole-derived phytoalexin camalexin also acts as a microbiome-modulating signal: in *Arabidopsis*, camalexin accumulation and exudation differed between interactions with beneficial and pathogenic bacteria, and camalexin further influenced *P. simiae* WCS417 by altering bacterial motility and chemotaxis (Koprivova et al. 2023, Zhou et al. 2025). More broadly, maize benzoxazinoids strongly shaped root and rhizosphere microbiota under field conditions (Cadot et al. 2021), and the volatile linalool was recently shown to induce the benzoxazinoid HDMBOA-Glc, which restructured the rhizosphere microbiome and enhanced resistance in subsequent plants (Guo et al. 2025).

Terpenoids and sterol-related metabolites likewise function as ecological filters in the rhizosphere. In melon, the triterpenoid cucurbitacin B, actively exported into soil by MATE (multidrug and toxic compound extrusion) transporters, enriched *Enterobacter* and *Bacillus* and increased resistance to *Fusarium oxysporum* (Zhong et al. 2022). In tomato, perturbation of the cycloartenol-derived triterpenoid pathway altered levels of β-sitosterol, stigmasterol, cholesterol, steroidal glycoalkaloids, and steroidal saponins, which correlated with shifts in bacterial families such as *Comamonadaceae* and *Sphingomonadaceae* (Guerrieri et al. 2025), consistent with broader evidence that phytosterols contribute to plant−microbe interactions (Der et al. 2024). Glucosinolates also play major microbiome-shaping roles: indole glucosinolates and their hydrolysis products altered rhizoplane, endosphere, and rhizosphere communities in *Arabidopsis*, with effects depending on both biosynthesis and breakdown pathways (Basak et al. 2024, Chroston et al. 2024, Acuña et al. 2025). In turn, bacterial colonization can depend on tolerance mechanisms against glucosinolate breakdown products, such as specific efflux pumps (Russ et al. 2025), while glucosinolate and defense-signaling mutants exhibit broad microbiome shifts (Kudjordjie et al. 2021). Finally, plant-derived small peptides are emerging as additional regulators of plant-associated microbiomes. These peptides can affect microbial gene expression directly or act indirectly through immunity and exudation patterns (Tan et al. 2024), and a tomato prosystemin-derived peptide was recently shown to restructure the phyllosphere microbiome, enriching genera such as *Sphingobium, Sphingomonas, Massilia*, and *Brevundimonas*, which are commonly associated with plant defense and resilience (Castaldia et al. 2025).

In addition to specialized metabolites, primary metabolites released through root exudation can play a central role in shaping rhizosphere microbiomes by creating selective nutritional niches that favour beneficial microorganisms over pathogens (Table 2). Amino acids are key drivers of microbial recruitment and activity. For example, glutamic acid has been shown to increase microbial abundance and selectively enrich taxa such as *Streptomyces*, highlighting its role as a modulator of microbiome structure (Kim and Kwak 2022). Similarly, glutamine acts as a strong chemoattractant and growth substrate for rhizobacteria, with its leakage regulated by the Casparian strip, thereby controlling spatial colonization patterns and preventing excessive microbial proliferation (Tsai et al. 2025). Under nutrient limitation, plants increase the exudation of organic acids such as citrate, malate, and oxalate, which enhance nutrient solubilization and shape rhizosphere microbial communities (Cesco et al. 2026). Organic acids further contribute to microbiome assembly. In rice, increased exudation of succinic acid and methylmalonic acid, regulated by the transcription factor OsPHR2, selectively stimulated *Pseudomonas* spp., enhancing phosphorus uptake and demonstrating a direct link between nutrient signaling, metabolite release, and beneficial microbiome recruitment (Liu et al. 2024).

Sugars and related compounds function as “prebiotic” metabolites that selectively support commensal microorganisms. A study in tomato identified compounds such as ribose, xylose, mannose, maltose, lactic acid, gluconolactone, and ribitol as preferentially utilized by beneficial bacteria but not by the pathogen *R. solanacearum*, enabling competitive exclusion and disease suppression (Wen et al. 2023). Similarly, variation in sucrose and trehalose levels across maize genotypes significantly shaped rhizobacterial communities across developmental stages, demonstrating that sugar composition is a key determinant of microbiome assembly (Lopes et al. 2022). Importantly, plant stress and aboveground signals can dynamically reprogram exudation patterns: foliar infection increased the release of organic acids, sugars, and amino acids, which simultaneously suppressed pathogens and enriched beneficial taxa such as *Trichoderma, Bacillus*, and *Streptomyces*, effectively reversing negative plant–soil feedback (Luo et al. 2022).

Beyond carbon metabolites, plant-derived vitamins and fatty acids also contribute to microbiome modulation. The vitamin riboflavin, together with the flavonoid 3-hydroxyflavone, was shown to restore disease-suppressive tomato microbiomes by enriching *Actinomycetota*, particularly *Streptomyces*, and indirectly reducing pathogen abundance, indicating that combinations of exuded metabolites can function as “prebiotic” signals for suppressive microbiome assembly (Yang et al. 2023a). Furthermore, developmental regulation can influence metabolite-mediated microbiome assembly: suppression of reproductive growth increased the rhizospheric exudation of fatty acids such as palmitic, oleic, linoleic, and stearic acid, which selectively enriched the beneficial fungus *Mortierella* and enhanced plant resilience and productivity (Hu et al. 2026). Together, these studies highlight that primary metabolites act not only as nutrients but also as key ecological drivers that structure microbial communities and promote soil suppressiveness (Table 2). Beyond effects on community composition, some primary metabolites directly regulate microbial recruitment behavior: root-derived sucrose stimulated levan production and flagellar synthesis in *Bacillus*, whereas inositol enhanced swimming motility in *Pseudomonas*, illustrating how exudates influence not only which microorganisms are favored, but also how efficiently they colonize the rhizosphere (Tian et al. 2021, O’Banion et al. 2023, Sánchez-Gil et al. 2023).

Collectively, these studies showed that plants actively shape their microbiomes through metabolite-mediated selection, often creating niches that favor beneficial microorganisms while limiting pathogens. Harnessing these processes offers opportunities to design crops and management strategies that steer the native microbiome via endogenous metabolites, complementing microbial inoculants, and enhancing agricultural resilience.

## Agricultural management strategies that shape microbiomes

Industrial agriculture has contributed to environmental degradation, including biodiversity loss, soil fertility decline, and climate change (Soria-Lopez et al. 2023). In response, policy frameworks such as the European directives on Soil Monitoring Law and Soil Strategy for 2030 aim to promote sustainable agronomic practices that reduce inputs while preserving or restoring ecosystem services (European Parliament 2026). In this context, the plant microbiome represents a key lever for achieving sustainable and resilient crop production (Wang et al. 2025b).

Many agroecological practices, such as reduced tillage, crop rotation, intercropping, cover cropping, and organic fertilization and amendments, can be viewed as ecosystem-level microbiome modulation strategies (Table 3). These approaches, historically embedded in traditional farming systems, are now increasingly understood through the lens of microbial ecology and multiomics as drivers of microbial diversity, community assembly, and ecosystem functioning (Bohan et al. 2022, Harwood 2024, Kim et al. 2025). Mechanistically, many are able to reshape resource availability, root-derived metabolites, and trophic interactions, thereby influencing microbiome composition, stability, and function.

**Table 3:** Impact of agricultural management strategies and practices on plant performances and microbiome structure and functionality to feed positive plant-soil feedback.

Agricultural management strategy and practice	Plant species/system	Plant performance outcome	Impact on microbiome (structure/function) and plant metabolome	Reference(s)
**Tillage**	Winter wheat	Reduced tillage practice and nitrogen fertilization intensity improved plant performance (e.g. highest total root length, shoot dry mass, root dry mass, fine root length) compared with the other practices in winter wheat	Enriched beneficial taxa (e.g. *Bacillus, Devosia*, and *Trichoderma*) Increased functional traits (e.g. siderophore production, trehalose synthase, and 1-aminocyclopropane-1-carboxylate deaminase) Enhanced plant metabolites (e.g. benzoxazinoid)	Behr et al. (2024)
**Tillage**	Wheat subjected to three different tillage treatments (plow, chisel plow, no tillage). Plant analysis at three different plant life stages	Higher rate of nitrogen uptake and relative growth rate were affected by tillage practices	Altered fungal diversity and rhizosphere colonization patterns AMF reduced under conventional tillage Saprophytic fungi enriched under conservation tillage, linked to plant growth	Li et al. (2021b)
**Crop rotation**	Peanut rotation system with maize, potato, and soybean	More efficient control of root rot disease caused by *F. oxysporum* (by 45%–56%) compared with monocropped regime	Enriched disease-suppressive bacterial taxa (e.g. *Paenibacillus, Pantoea*) Enhanced antimicrobial activity (volatiles, antibiotics) DMC restored pathogen suppression in monoculture	Zhou et al. (2023a)
**Crop rotation**	Four rotation systems for winter wheat with increased crop diversification (with spring maize and soybean) and reduction in intensive management practices	Higher average wheat yield; Legacy effect for the subsequent maize cultivation in the soil conditioned by crop diversification: increased plant height	Shifted bacterial and fungal communities via soil property changes (carbon storage, pH, nitrogen content) Enriched beneficial taxa (e.g. *Bacillus, Trichoderma*) Reduced abundance of pathogens (e.g. *Fusarium*) In the positive legacy effect, positive correlation between maize height and *Podospora* spp., enriched by crop diversification	Hu et al. (2025a)
**Cover cropping**	Interrow cover cropping with phacelia (*Phacelia tanacetifolia* Benth.) and rye (*Secale cereale* L.) plant species in vineyard	Phacelia cover cropping improved vine pruning weight and trunk diameter	Improved soil structure and AMF associations Increased fungal biomass and beneficial taxa (e.g. *Pseudarthrobacter*) Reduced pathogen incidence	Rocha et al. (2025)
**Cover cropping**	Maize cultivated in greenhouse in pots with 27 different soils from Chinese farmlands. Pots were pre-cultivated with hairy vetch (*Vicia villosa* Roth.) and Italian ryegrass (*Lolium multiflorum* Lam.) as cover cropping treatments	Maize biomass was used as a proxy for plant growth; Legume cover crops (hairy vetch) showed no significant effect on maize biomass at 20 sites, significantly decreased maize biomass at 3 sites, and significantly increased at 4 sites; Graminae cover crops (Italian ryegrass) negatively affected maize growth in soils from 21 sites	Legume cover crops enriched growth-promoting taxa (*Nonomuraea, Nordella*, and *Pedomicrobium*) and reduced detrimental microbes (e.g. *Jatrophihabitans*, LWQ8, and *Methylovirgula*) Gramineae crops increased taxa negatively associated with plant growth (e.g. *Mycobacterium*) Shifts in decomposer fungi linked to biomass outcomes	Shi et al. (2025)
**Intercropping**	Peanut-maize intercropping, rotated with oilseed rape	Boosted peanut height, biomass, and yields by increasing the availability of ammonium, nitrite and phosphate in soil	Increased flavonoid and coumarin exudation Enriched rhizosphere taxa (e.g. *Chloroflexota, Gammaproteobacteria*) Supplements of flavonoid and coumarin compounds to rhizobia pure cultures enhanced the expression of *nod* genes in *Bradyrhizobium* N47 and in turn stimulated in the plant the expression of *SYMRK, CCaMK*, and *NIN*, genes involved in nodule organogenesis	Qiao et al. (2024)
**Intercropping**	Soybean intercropped with maize	Soybean height and productivity were statistically improved in intercropped management system compared to monocropping under different nitrogen-fertilization regimes.	Increased nitrogen cycling pathways involved in nitrification (*amoB* and *nxrA*), dissimilatory nitrate reduction (*nrfA*), and Anammox (*hzsBC*) Functional taxa *Candidatus Jettenia, Lysobacter, Steroidobacter* and *Zooglea* correlated with yield	Shu et al. (2024)
**Organic amendments**	Application of four different pig manure treatments (two high regimes, low and no manure) on corn monoculture	Acceleration in phosphorus cycling that promoted the formation of larger root system, increasing maize productivity	Increased AMF and saprophytic fungi Enhanced multitrophic interactions (protists, nematodes) Improved microbiome-driven phosphorus cycling	Jiang et al. (2020)
**Organic amendments**	Amendment of soybean monoculture with lime, lime and soybean straw, lime and cow manure, and crude chitin	Soil amendments increased soybean plant height, plant biomass and seed weight; Crude chitin reduced by 70% disease incidence and strongly promoted up to 30% soybean yields	Increased microbial diversity and antagonistic taxa (e.g. *Pantoea, Bacillus, Humicola, Tausonia*, and *Mortierella*) Enhanced nutrient cycling functions Enriched chitin-degrading and nutrient-solubilizing microbes	Fan et al. (2026)
**Bio-organic fertilizers**	Amendment of cucumber with two organic fertilizers containing cow manure-bran and cow manure-bran-edible fungus residues, supplemented with *Bacillus subtilis* and *Trichoderma harzianum*	Improved cucumber growth parameters and reduced *Fusarium* wilt incidence	Shifted bacterial and fungal community composition (e.g. *Bacillus, Chitinophaga, Flavobacterium, Pennicillium*, and *Stagonospora*) Reduced pathogen abundance (e.g. *Fusarium*) Amelioration of soil physicochemical properties and organic matter content	Yang et al. (2025b)
**Plant stimulators**	Amendment of tomato variety Zhongza 301 with a gradient of calcium (0–800 mg Ca kg^−1^ soil)	Promotion of tomato height and fresh weight at higher Ca treatments; No wilt symptoms caused by *R. solanacearum* at high Ca treatments through induction of the host antioxidant enzymes, oxidative damage markers, and regulation of hormone homeostasis	Reshaped rhizosphere microbiome (e.g. *Burkholderia, Dyella Herbaspirillum*, and *Rhodanobacter*) Altered root exudation (e.g. salicylic acid, sugars) Suppressed pathogen, promoted antagonistic bacteria	(Bi et al. 2025)

A defining feature of intensive agricultural systems is the depletion of soil microbial diversity due to continuous cropping and high input use (Peralta et al. 2018). These legacy effects can persist for years, altering microbial network connectivity and nutrient cycling, and constraining microbiome recovery (Trivedi et al. 2020, Romdhane et al. 2022). In contrast, reduced tillage practices can enhance beneficial plant−microbe interactions by preserving soil structure and minimizing disturbance. For example, long-term reduced tillage in winter wheat enriched plant growth-promoting and antagonistic taxa, enhanced microbial functional traits, and improved stress tolerance and root development (Behr et al. 2024, Table 3). By analyzing three different tillage treatments along winter wheat developmental stages, the abundance of AMF and fungal network connectivity were correlated to higher nitrogen uptake and improved plant growth (Li et al. 2021b). Through the accumulation of crop residues and dead roots, conservation tillage altered the rhizosphere dynamics, supporting saprophytic fungi and other beneficial microorganisms (Li et al. 2021b).

Crop diversification strategies, including rotation, cover cropping, and intercropping, further promote microbiome modulation by increasing microbial diversity and functional redundancy (Table 3). In rotation systems, enrichment of disease-suppressive microbiome functions can significantly reduce pathogen pressure. For example, a DMC assembled from bacterial taxa associated with rotation-managed soils restored *F. oxysporum* suppression that had been lost under peanut monoculture (Zhou et al. 2023a). These effects may be largely mediated by shifts in root exudation patterns, which act as chemical drivers of microbiome assembly. For instance, garlic-derived metabolites in rotation systems reshaped the fungal communities (favoring reactive oxygen species-tolerant strains like *Penicillium* sp.) and suppressed pepper blight disease caused by *P. capsici*, while cover crops induce species-specific exudation profiles that alter microbial metabolic functions related to plant hormones (auxin and gibberellin) and enhance subsequent crop performance (Seitz et al. 2024, Wu et al. 2025a). Beyond pathogen suppression, crop rotation can also regulate plant−microbe−fauna interactions for pest control. In rice, rotation with multiple cover crops enhanced suppression of root-knot nematodes, with ryegrass showing the strongest effect. This was associated with the enrichment of antagonistic bacteria, including *Ramlibacter* and *Pseudomonas*, which reduced nematode infection by limiting gall formation and root penetration. Moreover, these rotation-induced microbiomes were shown to influence plant phytohormone balance, activating jasmonate-mediated defense pathways and thereby strengthening plant immunity (Zhang et al. 2025a). Importantly, increased crop diversification (rotation with multiple crops and reduced management intensity) not only built a beneficial rhizosphere microbiome in winter wheat, enhancing crop performance, but also established positive legacies for the subsequent cultivation of maize in soil pre-conditioned by crop diversification (Hu et al. 2025b).

Intercropping systems similarly drive microbiome modulation through coordinated changes in rhizodeposition and nutrient cycling (Table 3). For example, in peanut–maize intercropping systems rotated with oilseed rape, peanut roots increased the secretion of flavonoids and coumarins, which were associated with shifts in rhizosphere microbial communities, including enrichment of *Chloroflexota* and *Gammaproteobacteria* (Qiao et al. 2024). These metabolites appear to mediate chemical communication between plant roots and nitrogen-fixing bacteria, as demonstrated by the induction of nodulation genes in *Bradyrhizobium* and the activation of plant symbiosis-related pathways. This enhanced plant−microbe interaction translated into enhanced expression of enzymes involved in nitrogen cycling (Shu et al. 2024), improved nodulation, plant growth, and yield compared to monoculture systems. Beyond nitrogen fixation, crop diversification also increased soil nutrient availability, further supporting plant productivity. More broadly, these systems can promote complementary resource use, improve soil structure, and enhance microbial-mediated nutrient cycling (Li et al. 2021a).

Fertilization practices also play a central role in shaping microbiome dynamics (Table 3). While excessive agrochemical inputs can negatively impact biodiversity and increase disease susceptibility (Lekberg et al. 2021, Sabir et al. 2021), organic amendments (e.g. straw/manure/vermicompost applications) can restore soil carbon, improve soil physico-chemical features, and stimulate beneficial microbial interactions (Tao et al. 2020, Zhu et al. 2026). In banana, for example, organic fertilization (chicken manure) reduced *Fusarium* incidence by promoting protist grazing that enriched pathogen-suppressive *Bacillus* producing antimicrobial compounds (Guo et al. 2022). Similarly, crude chitin amendment improved soybean cultivation in monoculture by increasing microbial diversity and enriching for chitin degraders like the antagonistic taxa *Pantoea* and *Bacillus*, contributing to strongly reduce disease incidence (Fan et al. 2026). Calcium amendment in tomato enhanced plant height and fresh weight and prevented *R. solanacearum*-induced wilt at higher doses. The resistance was partly explained by altered root exudation (e.g. salicylic acid and sugars), along with increased antioxidant activity, improved hormone balance, and a reshaped rhizosphere microbiome enriched in beneficial taxa, collectively suppressing the pathogen and promoting antagonistic bacteria (Bi et al. 2025). A related approach involves the use of bio-organic fertilizer, which combine organic substrates with beneficial microorganisms. For example, a bio-organic fertilizer amended with *Bacillus amyloliquefaciens* led to the suppression of *Fusarium* wilt disease in banana monocropping. Plant protection was achieved through *B. amyloliquefaciens* direct inhibition of the pathogen and indirectly by increasing synergistic interactions with native *Pseudomonas* spp. (Tao et al. 2020). Besides reduced wilt incidence triggered by a shift in the rhizosphere composition, a bio-organic fertilizer composed of cow manure, *B. subtilis* and *T. harzianum* improved soil physico-chemical features, further boosting cucumber growth and health (Yang et al. 2025b). In addition, microbial inoculation strategies can complement sustainable farming practices. Under long-term organic farming, the application of DMC improved ryegrass growth potentially by enhancing nutrient acquisition, while reshaping the rhizosphere microbiome and enriching plant growth-promoting microorganisms (Behr et al. 2023). Notably, bio-organic fertilization can induce multitrophic effects, where interactions between microorganisms and soil fauna enhance pathogen suppression. For example, in tomato, bio-organic fertilization increased bacterivorous nematodes, which selectively enriched antagonistic bacteria (e.g. *Bacillus, Lysinibacillus*, and *Fictibacillus*) and their biocontrol metabolites, thereby reducing bacterial wilt (Xu et al. 2024).

Importantly, combining multiple sustainable practices can generate synergistic effects. A recent meta-analysis demonstrated that integrating diverse management strategies enhances agroecosystem services in cereal systems (Medrano et al. 2025). Similarly, combining reduced phosphorus fertilizer inputs with phosphorus-solubilizing microorganisms (e.g. *Paenibacillus polymyxa, B. amyloliquefaciens*, and *B. subtilis*) improves phosphorus availability and maize performance in degraded soils, highlighting the potential of integrated approaches to optimize microbiome function (Christel et al. 2021, Macik et al. 2023).

In summary, agroecological practices act as powerful drivers of microbiome modulation by reshaping microbial diversity, metabolic niches, and multitrophic interactions. However, the mechanisms underlying optimal combinations of these practices remain poorly understood. Developing predictive, systems-level frameworks that integrate ecological theory, multiomics data, and field validation will be essential to harness microbiome modulation for sustainable crop management.

## Toward an integrated approach to advance the understanding of microbiome modulation

The integrated approach of experimental, *in silico* modeling (e.g. genome-scale metabolic modeling), and multiomics (combining shotgun metagenomics, metabolomics, and transcriptomics) are increasingly used to provide high-resolution mechanistic understanding into (i) the complex interaction between beneficial microbial inoculants, soil fauna, and pathogens with the native microbiome, and the cascading impact on plant growth and health and (ii) genes, pathways, and metabolites mediating the disease suppression and abiotic stress mitigation of the modulated microbiome.

A growing body of work applying these integrative frameworks has begun to reveal causal links between microbiome modulation and enhanced plant performance (Table 4). Deng et al. (2022) demonstrated that bio-organic fertilizer, containing *B. amyloliquefaciens* T-5 combined with an organic amendment (rapeseed meal and chicken manure), induced suppressiveness against tomato bacterial wilt through microbiome modulation. Using metagenomics and cultivation-based approaches, the authors showed that members of the families *Sphingomonadaceae* and *Xanthomonadaceae*, capable of producing secondary metabolites, were specifically enriched in the rhizosphere following pathogen invasion. Experimental validation further confirmed that inoculation of isolates from these families into conducive soil significantly reduced pathogen abundance and increased the abundance of nonribosomal peptide synthetase genes, linking microbial community shifts to enhanced antimicrobial potential. In another study, Tao et al. (2023) demonstrated that bio-organic fertilizer, containing *Trichoderma guizhouense* NJAU4742 combined with an organic amendment, modified the composition and functioning of the native soil fungal microbiome to suppress Fusarium wilt on banana. The authors showed that the disease suppression action of this biofertilizer was a combination of direct pathogen suppression and the indirect effects due to interactions with native fungi (e.g. *Humicola*) in resource competition with pathogen and in induction of plant systemic resistance. Zhang et al. (2025b) conducted a rhizosphere microbiome transfer experiment demonstrating that the biocontrol efficacy of *Trichoderma asperellum* FJ035 against cucumber Fusarium wilt was mediated through modulation of the native microbiome. Plants receiving unsterilized rhizosphere soil showed a 45% lower disease index and 61% higher fresh weight than those receiving sterilized soil. Amplicon sequencing and cultivation-based analyses further showed that FJ035 altered the composition and abundance of rhizosphere microbial communities, indicating that disease suppression was mediated by microbiome restructuring rather than direct antagonism alone. A multiomics analysis of sugarcane treated with a 4-member DMC revealed coordinated interactions between microorganisms and the plant, including upregulation of bacterial genes involved in chemotaxis, quorum sensing, and nutrient acquisition. Concurrently, the DMC reprogrammed plant transcription related to hormone signaling, stress responses, and nutrient transport, ultimately enhancing photosynthesis, growth, and stress tolerance (Khoiri et al. 2025).

**Table 4:** Mechanistic insights into microbiome modulation revealed by integrated (multi)-omics, experimental approaches, and/or *in silico* modeling

Microbial inoculant and/or soil fauna	Plant species/system	Plant performance outcomes	Impact on microbiome features (structure/function) and plant metabolome	Methodological approaches	Reference(s)
*Bacillus amyloliquefaciens* T-5 combined with an organic amendment	Tomato	Suppressed bacterial wilt disease (*Ralstonia solanacearum*)	Enriched bacterial taxa (e.g. *Sphingomonadaceae* and *Xanthomonadaceae*) Enhanced antimicrobial potential	Metagenomics; cultivation-based method; field and glasshouse experiments	Deng et al. (2022)
*Trichoderma guizhouense* NJAU4742 combined with an organic amendment	Banana	Suppressed Fusarium wilt (*Fusarium oxysporum* f. sp. *cubense*); increased plant growth	Enriched pathogen-suppressing fungi (*Humicola*)	Metabarcoding; qPCR; cultivation-based method; pot experiment	Tao et al. (2023)
*Trichoderma asperellum* FJ035 and 30 bacterial strains	Cucumber	Suppressed Fusarium wilt (*F. oxysporum* f. sp. *cucumerinum*); increased plant growth	Enriched bacterial taxa (e.g. *Pseudomonas, Sinomonas, Ensifer*, and *Paenibacillus*)	DMC; metabarcoding; microbiome transfer	Zhang et al. (2025b)
*Trichoderma harzianum* T19 and five *Bacillus* strains	Wheat	Enhanced resistance to Fusarium crown rot (*Fusarium pseudograminearum*); promoted plant growth	Enriched beneficial microorganisms, including antagonistic fungi (*Trichoderma* and *Chaetomium*) and plant growth-promoting bacteria (*Pseudomonas* and *Paenibacillus*) Enhanced the accumulation of defense-related metabolites (e.g. epi-jasmonic acid, allantoin, Nβ-acetyltryptamine, and dihydrodaidzein)	Metabolomics; DMC	Zhou et al. (2026)
*Herbaspirillum seropedicae* strain RAM10	Wheat	Not tested	Differential enrichment of microbial taxa (e.g. *Massilia, Brevundimonas*, and *Filobasidium*) with putative roles in both plant energy and nitrogen metabolism	Metabarcoding; metabolomics	Carril et al. (2025)
*Bacillus thuringiensis* NL-11	Alfalfa *(Medicago sativa* L.)	Promoted plant growth	Modified the rhizospheric keystone microbial taxa (*Bacillota*) Influenced the metabolism of phenylpropanoids and isoflavonoids	Metabarcoding; metabolomics; pot experiments	Nie et al. (2025)
*Rhizobium pusense* TYQ1 and TYQ1-centered DMCs (7 bacterial strains)	Cucumber	Suppressed root-knot nematode infections (*Meloidogyne incognita*)	Enriched bacterial taxa (e.g. *Sphingomonas* and *Acidovorax*)	DMCs; metabolite feeding experiments; metabarcoding, transcriptomics; metabolomics; pot experiment	Liu et al. (2026a)
*Trichoderma spirale* RS05, *Bacillus velezensis* BD2231, and *Streptomyces mirabilis* BD2233	Hybrid bamboo (*Bambusa pervariabilis* × *Dendrocalamopsis grandis*)	Suppressed basal rot (*Fusarium proliferatum*); increased yield of bamboo shoots	Enriched bacterial taxa (e.g. *Agrobacterium, Burkholderia, Ochrobactrum, Achromobacter*, and *Azotobacter*) Stimulated the secretion of defense-related metabolites (e.g. trehalose-6-phosphate, berberine, chlorogenic acid, and piceatannol biosynthesis)	DMC; metabolomics; qPCR	Li et al. (2025b)
12 *Streptomyces* strains and 100-member bacterial DMC	Tomato	Suppressed bacterial wilt disease (*R. solanacearum*)	Promoted two native keystone taxa, *Stenotrophomonas maltophilia* CSC98 and *Paenibacillus cellulositrophicus* CSC13. CSC13’s metabolites induced the production of erythromycin E in *Streptomyces* R02	DMC; metabarcoding; transcriptomic; metabolomic; qPCR; pot experiments	Sun et al. (2026)
*Sphingomonas* sp. Hbc-6	Arabidopsis	Improve plant growth and drought resistance	Enriched beneficial bacterial taxa (e.g. *Streptomyces* and *Actinomycetospora*) Altered plant metabolites (amino acids)	Metabarcoding; metabolomics; cultivation-based method	Wang et al. (2024a)
*Bacillus*-containing biofertilizer	Tomato	Suppressed bacterial wilt disease (*R. solanacearum*)	Stimulated the growth of predatory protists (*Cercomonas* spp.) whose grazing activity in turn promoted *Bacillus* proliferation Upregulated functional polyketide synthase genes	Metabarcoding; metagenomics; qPCR; field, greenhouse, and pot experiments	Pei et al. (2026)
Predatory protists (*Naegleria* and *Cercomonas*) and 7-member bacterial DMC	Tomato	Suppressed bacterial wilt disease (*R. solanacearum*)	Enriched *Bacillus* populations, able to produce biofilms Upregulated associated genes (e.g. *epsA-O, tapA-sipW-tasA operon*) and synthesized secondary metabolites (e.g. macrolactin H, bacillaene, and difficidin)	DMCs; metabarcoding; metatranscriptomics, greenhouse studies and microcosm assays; bacterial mutant strains	Yue et al. (2025)
122-member bacterial DMCs (*Pseudomonadota, Bacteroidota, Bacillota*, and *Actinomycetota*) and nematodes (*Caenorhabditis elegans, Distolabrellus veechi, and Panagrolaimus* sp. NJ)	Tomato	Suppressed bacterial wilt disease (*R. solanacearum*)	Enriched *Pseudomonadota* species (e.g. *Klebsiella michiganensis* and *Raoultella ornithinolytica*)	DMCs; genomics, metatranscriptomics, field studies and microcosm assays	Li et al. (2026a)
122-member bacterial DMCs and protists (*Naegleria gruberi,Vahlkampfia inornata, Dimastigella trypaniformis*, and *Colpoda aspera*)	Tomato	Suppressed bacterial wilt disease (*R. solanacearum*)	Enriched high-GC, acid-preferring bacteria and metabolite exchange Upregulated key plant growth-promoting pathways	DMCs; metabarcoding; metagenomics; metaranscriptomics; genome-scale metabolic models	Liu et al. (2026b)
122-member bacterial DMCs and nematodes (*Distolabrellus veechi* and *Panagrolaimus* sp.)	Tomato	Suppressed bacterial wilt disease (*R. solanacearum*)	Enriched *Pseudomonadota* (*Alphaproteobacteria*)	DMCs; metabarcoding; pot experiment	Chuai et al. (2026)

An integrated approach combining microbiome, metabolome, and soil enzyme analyses with DMCs has proven crucial for understanding not only whether and how microbial inoculants modulate the microbiome, but also how they influence plant metabolic homeostasis. For example, Zhou et al. (2026) showed that DMC application enhanced wheat growth and yield while suppressing Fusarium crown rot by optimizing rhizosphere physicochemical conditions, restructuring microbial communities, and reestablishing metabolic homeostasis. Their analyses revealed strong correlations between DMC-induced microbial and biochemical shifts and improved plant health, including the accumulation of defense-related metabolites such as epi-jasmonic acid, allantoin, Nβ-acetyltryptamine, and dihydrodaidzein. Similarly, the plant growth-promoting bacterium *Herbaspirillum seropedicae* RAM10 has been identified as a catalyst of microbiome modulation and metabolic reprogramming in wheat, enriching bacterial taxa within *Pseudomonadota* and *Actinomycetota* associated with plant energy and nitrogen metabolism, and altering the abundance of metabolites related to phenylpropanoids, terpenoids, and unsaturated fatty acids (Carril et al. 2025). A cross-kingdom DMCs enhanced bamboo growth and suppressed basal rot disease through synergistic enhancement of microbiome modulation and plant immunity priming. DMCs stimulated plants and microorganisms to produce more defense-related metabolites (e.g. trehalose-6-phosphate, berberine, chlorogenic acid, and piceatannol biosynthesis) against pathogen invasion and induced plant systemic resistance (Li et al. 2025b). However, transcriptomics and proteomic analyses, combined with targeted metabolite supplementation and mutant-based assays are still needed to establish direct causal relationships between DMC-mediated metabolic shifts and host resistance. In this context, Sun et al. (2026) recently showed that the biocontrol efficacy of *Streptomyces* spp. was associated with shifts in the rhizosphere microbiome, particularly the promotion of two native keystone taxa, *S. maltophilia* CSC98 and *Paenibacillus cellulositrophicus* CSC13. Transcriptomic and metabolomic analyses revealed that CSC13’s metabolites induced the production of erythromycin E in *Streptomyces* R02, a key compound that directly suppressed tomato bacterial wilt disease.

Accumulating evidence highlights the ecological roles of faunal−microbial interactions on plant health (Table 4). Using a long-term field study complemented by greenhouse and pot experiments, Pei et al. (2026) demonstrated successful microbiome modulation by harnessing protistan predation to enhance suppression of tomato bacterial wilt. Application of a *Bacillus*-containing bio-organic fertilizer significantly stimulated the growth of *Cercomonas* spp., whose grazing activity in turn promoted *Bacillus* proliferation and upregulated functional polyketide synthase genes, ultimately increasing pathogen inhibition. Through a combination of DMCs with genomics, metatranscriptomics, field studies and microcosm assays, Li et al. (2026a) highlighted the importance of multitrophic interactions between microbivorous nematodes and bacteria in shaping the emergent microbiome functions that influence plant health. The authors demonstrated that nematode predation suppressed dominant bacterial taxa and promoted the proliferation of less abundant but functionally relevant species, thereby enhancing microbial traits such as carbon substrate utilization and antibiotic production, ultimately leading to the suppression of *R. solanacearum*. Furthermore, the disease suppressive effect of *Klebsiella michiganensis* and *Raoultella ornithinolytica* were amplified in the presence of nematodes, indicating that predation can not only restructures the microbiome but also facilitates complex, beneficial interspecies interactions (Li et al. 2026a). In a pivotal study, Liu et al. (2026b) investigated how protist predation can shift bacterial metabolic interaction and functional potential by integrating continental-scale analysis of 757 soil samples, *in silico* modeling, and controlled experiments. The authors demonstrated that protist predation preferentially reduced low genomic (guanine-cytosine, GC content), sugar-preferring taxa while enriching high-GC, acidophilic bacteria. DMC validation experiments further showed that protist predation sustained the exchange of organic and amino acid metabolites (e.g. L-leucine, L-valine, L-cysteine, and formate) within bacterial communities, indicating enhanced metabolic cross-feeding among surviving taxa. These metabolic reorganizations increased interaction potential within bacterial communities, promoted the expression of plant growth-promoting pathways (e.g. IAA and siderophore production), and ultimately improved tomato height and biomass.

## Emerging tools and strategies for next-generation microbiome modulation

### Function-oriented multitrophic DMCs

Trait-based ecological frameworks provide a foundation for advancing our understanding of the functional basis of plant−microbiome interactions. Such frameworks enable the rational design of microbiome assemblies based on functional traits rather than solely taxonomic composition. For example, a trait-based multicomponent framework has been proposed to guide the challenging task of optimal DMC design. In this context, identifying microbial keystone taxa, which are specific members of the microbiome exerting a disproportionately large impact on ecosystem structure, stability, and function relative to their abundance, may provide a practical strategy to support rational DMCs design (Espíndola-Hernández et al. 2026).

There is a growing interest in expanding the design of DMCs from single trophic-level systems focused on predefined functions to multitrophic systems that integrate ecological interactions across the phytobiome. Such complex micro-food web-based communities are expected to exhibit greater stability, functional redundancy, and resilience (Luan et al. 2025). In this context, multitrophic DMCs can be defined as microbial communities assembled based on ecological traits and interactions across multiple trophic levels, enabling their establishment and function within the phytobiome (Luan et al. 2025). However, a major challenge has been predicting and inferring trophic interactions between multiple species especially at a microbial level (Li et al. 2024c). A step toward addressing this is through the development of trait-based ecological frameworks, which provide a strong basis for the rational design of multitrophic DMCs (Luan et al. 2025, Liu et al. 2026b). A recent framework based on fungal genomics demonstrated that traits such as genome size and guanine-cytosine (GC) content can capture trade-offs among growth, nutrient acquisition, and stress tolerance, which mediate plant productivity (Chen et al. 2025a). Furthermore, genomic traits, including secondary metabolite gene clusters, can be used to predict bacterial susceptibility to nematode predation (i.e. palatability) (Li et al. 2024c). Such a framework demonstrates that predator−prey interactions can be inferred directly from genomic data, enabling the rational design of multitrophic DMCs by selecting taxa that are well integrated into native trophic networks. For example, DMC enriched in predator-preferred taxa such as *Pseudomonadota*, rather than commonly used *Bacillota* that are less connected to trophic interactions, can activate soil food web processes, enhance pathogen suppression, and reduce reliance on chemical inputs (Li et al. 2026a, Liu et al. 2026b).

Overall, the complex interactions between plants, soil, and native microbiomes presents a significant challenge in the development of function-oriented multitrophic DMCs that is safe and effective (Garcés-Ruiz et al. 2026). In this context, integrating *in silico* predictions, including artificial intelligence and machine learning, genome-scale metabolic models and dynamic flux balance analysis, with experimental data (including field studies) and multiomics datasets may enable the rational design of safe and effective DMCs. Such framework can (i) predict and optimize DMC compatibility, stability, and field performance based on (meta)genomic, transcriptomic, and phenotypic data, (ii) identify potential safety risks, including antibiotic resistance and toxin production, (iii) assess environmental persistence, and (iv) support decision-making through interpretable models (Portal-Gonzalez et al. 2025, Garcés-Ruiz et al. 2026).

### Temperate phages

Although temperate phages are traditionally considered unsuitable for agricultural application due to their lysogenic lifestyle (as they can disseminate virulence and antibiotic resistance genes), emerging evidence suggest their potential to support beneficial microorganisms (Salehimoghaddam et al. 2026). Dougherty et al. (2023) revealed widespread and largely uncharacterized prophage activity, diversity, and function in phyllosphere-associated bacteria, including *Erwinia* and *Pseudomonas*. Through Virion Induction Profiling Sequencing, the authors identified and quantified the activity of 120 spontaneously induced prophages combined with *in planta* experiment and showed that prophages in *Erwinia aphidicola* contribute substantially to intraspecies genetic diversity, partitioning bacterial populations into antagonistic factions engaged in microbial competition. These findings highlight the dual role of prophages in microbiome assembly and function: while some may suppress pathogenic strains and promote beneficial community establishment, others may potentially eliminate plant-beneficial bacteria or facilitate the spread of undesirable traits, although this remains poorly characterized in plant microbiome. Thus, temperate phages represent a promising yet complex component for designing bacteria-based DMCs, provided their ecological roles and risks are carefully considered. Furthermore, dual-function phage cocktails combining lytic and temperate phages may provide a complementary strategy for microbiome modulation (Salehimoghaddam et al. 2026). By leveraging phage lifestyle diversity, such cocktails may broaden pathogen coverage, reduce the emergence of phage resistance, and maintain greater ecological safety than individual broad host-range phages. In particular, temperate phages within these cocktails may influence bacterial fitness, gene expression, and stress tolerance, as well as community interactions, ultimately reshaping microbiome structure and function (Salehimoghaddam et al. 2026).

The broader implementation of phage-based microbiome modulation strategies, including both lytic and temperate phages, remains constrained by several challenges including industrial scaling, field performance, safety consideration, heterogeneous regulatory guidelines and approval frameworks across countries, many of which are still-evolving (Czajkowski et al. 2025, Darriaut et al. 2025). In particular, phage cocktails may require frequent updates to maintain efficacy against evolving pathogens, while current EU regulations often require re-registration following formulation changes, limiting flexibility and increasing commercialization costs (Toussaint et al. 2024). In addition, the ecological complexity of temperate phages, including their potential role as reservoir of antibiotic resistance genes and toxin genes, poses further challenges for environmental risk assessment and regulatory approval (Czajkowski et al. 2025).

### Microbiome transplantation and breeding

Microbiome composition (and thus function) can be altered independently of the host genome through microbiome transplantation. This approach involves transferring microbial communities from a donor system with desirable functions to a recipient, typically over short ecological timescales. In contrast, microbiome breeding represents an iterative extension of transplantation that operates over longer, evolutionary timescales by selecting microbiomes based on their effects on host performance (Shi et al. 2026).

Microbiome transplantation has been widely demonstrated in human systems, such as faecal microbiota transplantation for *Clostridium difficile* infections (Allegretti et al. 2019), and is increasingly explored in plants. In plant systems, microbiome transfer can confer beneficial traits, including disease suppression, enhanced nutrient acquisition, and improved tolerance to abiotic stresses (Mendes et al. 2011, Jiang et al. 2022, Zhang et al. 2022, Ehau-Taumaunu and Hockett 2023, Abou Jaoudé et al. 2025, Guilherme Pereira et al. 2025, De Zutter et al. 2021). Typically, a subset of donor microorganisms establishes within the recipient microbiome, leading to functional shifts that influence plant phenotype. However, outcomes are often context-dependent and can vary across host species and environments, highlighting challenges in predictability (Chen et al. 2025c).

Within greenhouse experiments, microbiome transplantation is most commonly achieved by introducing a fraction of donor soil (e.g. 10%–20% by weight) into recipient systems (Mendes et al. 2011, Jiang et al. 2022). However, moving such large volumes of donor soil are impractical for field application. Consequently, successful field-scale implementation may depend less on bulk soil transfer and more on identifying compatible donor-recipient microbiomes and key microbial taxa through large-scale microbiome mapping and functional characterization of agricultural soils, as demonstrated by recent nationwide microbiome initiatives in Denmark (Singleton et al. 2026). Such approaches could enable the targeted introduction of well adapted microbial taxa and functions with a greater likelihood of establishment and persistence in recipient environments (Zhou et al. 2024a, Jiang et al. 2022). In the phyllosphere, transplantation is typically performed by transferring leaf-associated communities via foliar sprays (Morella et al. 2020, Ehau-Taumaunu and Hockett 2023, Xu et al. 2026b). Phyllosphere microbiome transplantations are therefore much more compatible with existing agricultural practices and have been shown to improve yields (Mehlferber et al. 2023). They do still require large quantities of viable microbial biomass to be transferred, and often in repeated applications, due to the resilience of native communities. To improve transplantation efficiency, the exploration of taxa already adapted to the recipient phyllosphere environment is vital (Zhou et al. 2024a), since the phyllosphere represents a much more specialised environment compared to roots and soil, and thus only a narrow subset of plant-associated microbes can successfully colonize it (Barnes et al. 2026).

Another consideration of microbiome transplantation is the potential for unanticipated pathogen transfer, and the regulatory challenges of releasing complex microbial communities into the environment (Jiang et al. 2022, Darriaut et al. 2025). Furthermore, as with DMCs, the ecological stability, persistence, and potential long-term impacts of transplanted microbiomes on native communities, as well as microbiome-mediate functions such as pest and disease control, remain important considerations for environmental risk assessment and regulatory approval (Xu et al. 2025, Garcés-Ruiz et al. 2026). With in depth multiomic analyses, it is increasingly possible to characterize microbial communities in depth, and also gain mechanistic insight into plant−microbe and microbe−microbe interactions. These methods are becoming increasingly well established and affordable. Consequently, it is possible to screen out many microorganisms with likely harmful ecological or health effects (to human or plants) in exploratory phases, while more effectively identifying beneficial microorganisms for transplantation from field conditions (Garcés-Ruiz et al. 2026).

### Microbiome breeding

Microbiome breeding is an iterative subset of microbiome transplantation that operates over evolutionary timescales (Bell et al. 2025). In this approach, the microbiome is treated as a selectable trait that can be optimized for desired effects on plant phenotype, analogous to host breeding. Typically, the process begins with sourcing a complex environmental microbiome with diverse taxonomic and functional composition (Morella et al. 2020, Bell et al. 2025). This initial inoculum should contain microorganisms with desirable beneficial traits. Plants are then grown with the microbiome under defined abiotic or biotic stress conditions. After a growth period, plant performance is assessed, and microbiomes from the top-performing plants are used as inocula for subsequent rounds of selection through iterative serial passaging. Over successive cycles, this process enriches for microbiomes that enhance plant performance under the target stress conditions (Mueller and Linksvayer 2022).

This iterative selection has been shown to improve plant performance, including enhanced tolerance to salinity and drought, as well as increased nutrient acquisition (Mueller et al. 2021, Faller et al. 2024, Angot et al. 2025, Guilherme Pereira et al. 2025). However, translating microbiome breeding to field conditions remains challenging, largely due to variability in environmental conditions and limited scalability. Conducting selection directly under field conditions may improve persistence and performance, although more selection cycles may be required. Further, integrating plant and microbiome breeding could enable co-optimization of host−microbe interactions (Gopal and Gupta 2016).

Breeding of phyllosphere microbiomes presents additional challenges, as these communities are more stochastic and less stable than root-associated microbiomes (Deng et al. 2024, Li et al. 2025a). For example, serial passaging of leaf microbiomes led to both suppressive and permissive states against bacterial leaf speck (Ehau-Taumaunu and Hockett 2023), highlighting their variability. Nevertheless, given their potential growth benefits and the relative ease of application (Mehlferber et al. 2023, Xu et al. 2026b), phyllosphere microbiomes remain a promising target for microbiome-based crop improvement.

### Functional synbiotics

As noted earlier, plant roots release primary and specialized metabolites that support microbial growth and activity. Nutritional niche availability is one of the key factors determining the successful proliferation of microbial inoculants within the native microbiome (Čaušević et al. 2024). Limited carbon niche availability and competition can restrict the proliferation of inoculants (e.g. *Pseudomonas*), which may further lose productivity through metabolite sharing with the native bacterial taxa (Čaušević et al. 2024). In contrast, providing inoculant-specific nutrient niche can improve their survival and facilitate functional integration into the native microbiome (Čaušević et al. 2024). This principle underpins “synbiotic” strategies, which combine beneficial microorganisms (probiotics) with selective metabolites, compounds, or nutrients (prebiotics) that support microbial growth or activity, and consequently improve microbiome-mediated effects on plant growth and health. For example, lipid prebiotics applied with a *Streptomyces* probiotic significantly improved the control of Fusarium root rot disease while preserving the native bacterial diversity, enhancing *Streptomyces* colonization and fostering cooperative interactions among beneficial bacteria taxa (You et al. 2026). Similarly, the combined application of low phosphorus-enriched metabolites (succinic acid, azelaic acid, threonic acid, and methionine) and key microbial taxa effectively suppressed cucumber Fusarium wilt even under high phosphorus conditions (Liu et al. 2026c). Moreover, amino acid prebiotics applied with *Pseudomonas* spp. probiotics exhibited the highest resistance to insect herbivory compared with a water control and with the individual application of amino acids or *Pseudomonas* sp. The amino acids served as nutrients and attractants, significantly boosted the growth and rhizosphere/root colonization of *Pseudomonas* spp. (Yang et al. 2025a). Furthermore, co-application of 2-naphthoic acid and *Sinorhizobium* sp. CS204 significantly improved plant nutrient acquisition and photosynthesis in soybean under drought (Chen et al. 2026). Alternatively, inoculants can cooperate with native microbiome members to expand their collective resource use. For example, *Sphingomonas* and *Rhizobium* strains, which occupy similar ecological niches when inoculated individually, can modulate each other’s proteome when co-inoculated, thereby extending their niche breadth and enabling the utilization of complementary, non-overlapping carbon sources (Hemmerle et al. 2022).

A complementary proof-of-concept strategy involves disrupting pathogen chemotaxis for reducing disease pressure. Here, Ji et al. (2025a) showed that the natural coumarin compound esculetin inhibits *Phytophthora sojae* chemotaxis by targeting its isoflavone receptor (IRK1 for genistein), while simultaneously enhancing *Rhizobium* activity and symbiosis through induction of nodulation-related genes and enhancing nitrogenase activity.

Overall, advances in understanding of the metabolic ecology of microbiomes, including nutrient augmentation, nutrient blocking, cross-species niche facilitation, and chemotaxis inhibition, offers powerful avenues for microbiome modulation and improved plant performance (Ji et al. 2025b, Bakkeren et al. 2025). Integrating these strategies with additional mechanisms such as phage or protist predation may further enhance pathogen suppression and microbiome functionality, as demonstrated in other host-associated microbiome systems (Lentsch et al. 2025).

## Concluding remarks, research gaps, and future perspectives

**Figure 1:**
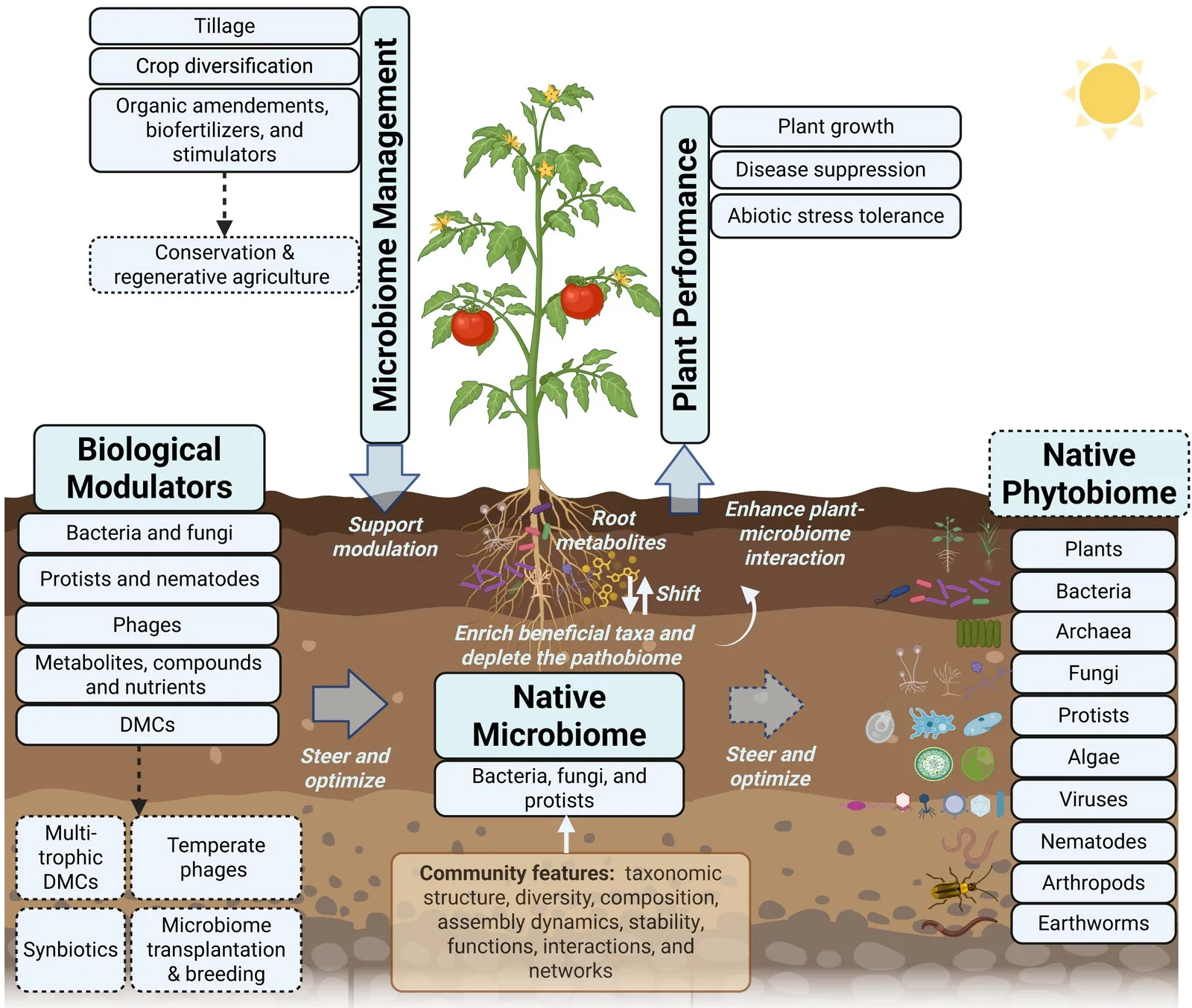
Conceptual framework of microbiome modulation for improving plant performance. The framework illustrates the transition from single-trophic-level or single-component approaches (solid boxes) to systems-level, integrated strategies (dashed boxes and black arrows) that leverage (agro-)ecological principles and multitrophic interactions. Biological modulators—including bacteria, fungi, protists, nematodes, phages, metabolites, compounds, nutrients, and DMCs—act to steer and optimize the native plant-associated microbiome (predominantly bacterial and fungal communities). These approaches are supported by agricultural management practices such as tillage, crop diversification, and the application of organic amendments, biofertilizers, and stimulators. Microbiome modulation operates by reshaping key community features, including taxonomic structure, diversity, composition, assembly dynamics, stability, functional capacity, interactions, and network architecture. An important component of this framework is the role of plant-derived root metabolites (primary and specialized), which drive shifts in microbiome composition and activity by acting as carbon sources, ecological filters, and signaling molecules in the rhizosphere. These metabolite-mediated changes regulate microbial recruitment, behavior, and interactions, thereby enhancing plant–microbiome interactions and further promoting the enrichment of beneficial taxa while depleting pathobiome members. Emerging tools and strategies (dashed boxes), including function-oriented multitrophic DMCs, temperate phages, microbiome transplantation and breeding, and functional synbiotics, further expand the capacity to modulate the microbiome for desired plant-microbiome beneficial outcomes. Future microbiome modulation strategies are expected to extend beyond the microbiome to the broader phytobiome, encompassing plants, bacteria, archaea, fungi, protists, algae, viruses, and soil fauna (e.g. nematodes, arthropods, and earthworms), thereby integrating multitrophic interactions across the soil–plant interface. Collectively, these processes contribute to improved plant performance, including enhanced growth, disease suppression, and tolerance to abiotic stress. Created in https://BioRender.com.

Microbiome modulation through varied approaches of biological interventions represents a paradigm shift in advancing sustainable and climate-resilient agri-food systems (Compant et al. 2025). Beyond their direct effect, diverse biological interventions, including inoculants of bacteria, fungi, protists, nematodes, phages, and metabolites, can steer and optimize the native microbiome to enhance plant growth, disease suppression, and abiotic stress tolerance (Fig. 1). These effects can be mediated through key ecological processes such as biodiversity function, community assembly dynamics, stability, competitive niche occupation (nutritional and physical), multitrophic interactions, ecological networks and micro-food web restructuring, and metabolic reprogramming. Microbiome-based applications operate across multiple ecological layers and therefore benefit from integrating complementary mechanisms rather than relying on single modes of action. For example, competitive niche exclusion can stabilize early colonization, metabolic reprogramming strengthens plant-microbe and microbe-microbe feedbacks, DMCs enhance functional redundancy and resilience, and protists, nematodes, and phages offer precision tools for targeted enrichment or removal of specific taxa. However, microbiome modulation does not always yield positive effects on plants (van Overbeek et al. 2025), highlighting the need for more predictive frameworks. In this context, the N + 1/N − 1 concept, which systematically evaluates the addition or removal of individual community members offers a powerful strategy to disentangle microbiome function. By integrating classical microbiology, -omics technologies, and modeling, these approaches enable mechanistic insights into community dynamics, supports structure-function relationships, and advance the development of robust and predictive microbiome modulation strategies (Burz et al. 2023). Beyond these approaches, integrating and harnessing ecological and evolutionary principles, linking biotic and abiotic interactions (ecology) with the evolutionary trajectories of plants and their associated microbiomes, will further improve our ability to predict and modulate the microbiome functions in agroecosystems, by for example using adaptive coevolution as a basis for designing microbiome-based stress management strategies (Morales Moreira et al. 2023, Delgado-Baquerizo et al. 2025). An additional and largely underexplored avenue for microbiome modulation lies in harnessing host genes that regulate plant−microbiome interactions. Emerging evidence indicates that microbiome-shaping (Ms) and microbiome-responding (Mr) genes can modulate microbiome assembly and function by influencing the recruitment and maintenance of beneficial microbial taxa and plant responses to microbiome-derived signals, thereby affecting microbiome-mediated functions such as disease suppression (Cernava 2024, Lv et al. 2024, Misu et al. 2025, Wang et al. 2025). Unlike conventional resistance (R) genes, which often confer pathogen-specific resistance and may involve growth-immunity trade-offs, Ms and Mr genes can enhance plant performance indirectly through microbiome restructuring and the enrichment of beneficial taxa (Lv et al. 2024, Wang et al. 2025). Integrating these genes into breeding programs may therefore offer new opportunities to develop more durable disease-resistant and climate-resilient crops by engineering the host capacity to recruit and maintain beneficial microbiomes. By deliberately shaping the microbiome features, microbiome modulation enables the development of next-generation bioproducts with dual benefits: direct effect on the plant or pathogen and indirect effects via the native microbiome (Hu et al. 2023, Darriaut et al. 2025, Salehimoghaddam et al. 2026). However, a deep understanding of the ecological processes and mechanistic drivers underlying microbiome modulation, along with the development of predictive models to guide agricultural management strategies and practices that shape microbiomes over time, remains essential. In particular, integrated agricultural management systems, including organic, conservation, and regenerative approaches, should be systematically evaluated for their long-term and combined effects on the microbiome and plant performance, as well as for their resilience under changing climatic conditions. Addressing these challenges requires integrated frameworks that combine experimental validation *in planta* (including long-term field studies) and reductionist approaches via DMCs with *in silico* modeling and multiomics analyses to better predict, design, and sustain beneficial plant-microbiome outcomes. Further progress will also depend on the development of enabling resources, including comprehensive databases and biobanks, alongside trait-based ecological frameworks to support the design of function-oriented multitrophic DMCs (Luan et al. 2025).

As the field progresses toward phytobiome-level understanding and modulation, future studies should routinely incorporate additional markers (e.g. 18S rRNA gene for eukaryotic microorganisms and mitochondrial Cox1 gene for soil fauna) within multigene metabarcoding frameworks, alongside traditional ones (e.g. 16S rRNA gene and ITS), as well as viromics (viral metagenomics) to better capture cascading effects on microbial, faunal, and viral communities. More broadly, advances in high-throughput sequencing technologies and declining computational and sequencing costs are increasingly making large-scale shotgun metagenomics feasible alternatives or complementary to targeted metabarcoding, enabling simultaneous taxonomic and functional characterization across the phytobiome. Recent large-scale studies further highlight the growing potential of these approaches to resolve complex microbiome structure and function at unprecedented depth and scale (Singleton et al. 2026). In this context, long-read sequencing technologies (e.g. Oxford Nanopore Technologies and Pacific Biosciences), which can generate long amplicons, including full-length markers or multigene operons, offer promising alternatives or complements to short-read Illumina platforms by improving taxonomic resolution (Cook et al. 2024, Stalder et al. 2025, Bista and Lino 2026). Given their ecological importance in agroecosystems, more attention should also be paid to archaeal (e.g. *Nitrososphaerota*) and algal communities and their responses to biological modulators (Lee and Ryu 2021, Kleinbölting et al. 2026). In addition, the role of other soil fauna, including arthropods (e.g. springtails) and earthworms, in shaping community features warrants closer attention, as their trophic interactions influences plant growth and health (Li et al. 2025c, Levasseur et al. 2025, Wang et al. 2026). For instance, recent evidence shows that soil fauna can actively modulate plant root exudation patterns, thereby indirectly shaping community assembly and function (Levasseur et al. 2025). At the same time, microorganisms themselves actively modify their environment (e.g. soil pH), highlighting the importance of bidirectional and dynamic microbe−environment interactions in determining plant performance (Wang et al. 2025a).

Finally, beyond phages, other narrow-spectrum antimicrobial strategies, such as bacterial and extracellular contractile injection systems, remain largely unexplored, despite their potential for precision manipulation of plant microbiomes (Madushani et al. 2026). Advances in synthetic biology offer new opportunities to control microbial behavior in complex plant environments through tools such as CRISPR interference, biosensor circuits, and quorum-sensing modules (Portal-Gonzalez et al. 2025). For example, engineered bacteria expressing surface-displayed polysaccharide-recognizing proteins have been shown to enhance root colonization by nitrogen-fixing bacteria, increasing the abundance of beneficial taxa (*Rhizobiales* and *Sphingomonadales*) and promoting plant growth (Liu et al. 2026d). However, regulatory and biosafety concerns due to engineering of the bacteria remain significant barriers to field deployment. The development of containment strategies, such as genetic kill switches and nutrient-dependent circuits, may therefore advance the application of microbiome modulation strategies involving engineered microorganisms in agriculture (Portal-Gonzalez et al. 2025).

## Data Availability

No data was used for the research described in the article.
